# A Smartphone-Based Pain Management App for Adolescents With Cancer: Establishing System Requirements and a Pain Care Algorithm Based on Literature Review, Interviews, and Consensus

**DOI:** 10.2196/resprot.3041

**Published:** 2014-03-19

**Authors:** Lindsay A Jibb, Bonnie J Stevens, Paul C Nathan, Emily Seto, Joseph A Cafazzo, Jennifer N Stinson

**Affiliations:** ^1^Hospital for Sick ChildrenToronto, ONCanada; ^2^Lawrence S Bloomberg Faculty of NursingUniversity of TorontoToronto, ONCanada; ^3^Institute of Health Policy, Management and EvaluationUniversity of TorontoToronto, ONCanada; ^4^Centre for Global eHealth InnovationUniversity Health NetworkToronto, ONCanada

**Keywords:** adolescent, pain, neoplasms, cellular phone, algorithms

## Abstract

**Background:**

Pain that occurs both within and outside of the hospital setting is a common and distressing problem for adolescents with cancer. The use of smartphone technology may facilitate rapid, in-the-moment pain support for this population. To ensure the best possible pain management advice is given, evidence-based and expert-vetted care algorithms and system design features, which are designed using user-centered methods, are required.

**Objective:**

To develop the decision algorithm and system requirements that will inform the pain management advice provided by a real-time smartphone-based pain management app for adolescents with cancer.

**Methods:**

A systematic approach to algorithm development and system design was utilized. Initially, a comprehensive literature review was undertaken to understand the current body of knowledge pertaining to pediatric cancer pain management. A user-centered approach to development was used as the results of the review were disseminated to 15 international experts (clinicians, scientists, and a consumer) in pediatric pain, pediatric oncology and mHealth design, who participated in a 2-day consensus conference. This conference used nominal group technique to develop consensus on important pain inputs, pain management advice, and system design requirements. Using data generated at the conference, a prototype algorithm was developed. Iterative qualitative testing was conducted with adolescents with cancer, as well as pediatric oncology and pain health care providers to vet and refine the developed algorithm and system requirements for the real-time smartphone app.

**Results:**

The systematic literature review established the current state of research related to nonpharmacological pediatric cancer pain management. The 2-day consensus conference established which clinically important pain inputs by adolescents would require action (pain management advice) from the app, the appropriate advice the app should provide to adolescents in pain, and the functional requirements of the app. These results were used to build a detailed prototype algorithm capable of providing adolescents with pain management support based on their individual pain. Analysis of qualitative interviews with 9 multidisciplinary health care professionals and 10 adolescents resulted in 4 themes that helped to adapt the algorithm and requirements to the needs of adolescents. Specifically, themes were overall endorsement of the system, the need for a clinical expert, the need to individualize the system, and changes to the algorithm to improve potential clinical effectiveness.

**Conclusions:**

This study used a phased and user-centered approach to develop a pain management algorithm for adolescents with cancer and the system requirements of an associated app. The smartphone software is currently being created and subsequent work will focus on the usability, feasibility, and effectiveness testing of the app for adolescents with cancer pain.

## Introduction

### The Burden of Pain in Adolescents With Cancer

Pain is one of the most distressing symptoms in adolescents with cancer. It is estimated that 49%-62% of adolescents with cancer will experience pain related to the disease and/or associated invasive procedures and treatments [1-3]. Pain negatively affects an adolescent’s quality of life [3-5], impedes cancer recovery [6], results in adolescent and family distress [7-10], and is associated with long-term morbidity [11,12]. Pain related to cancer also represents a significant cost burden to the health care system and families [13], with pain being the most common reason for cancer patients to utilize emergency health services [14-16].

Treatment advancements and health care system changes have allowed adolescents with cancer to spend increasing amounts of time in the outpatient setting over the course of their cancer care [17,18]. While this is a welcome change for many adolescents and families, it means that a significant amount of cancer symptoms (including pain) are experienced in an environment where management options may be limited (eg, at home). Despite the impact of cancer on adolescent health quality and cost, very limited research has been conducted on how to best manage outpatient pain in adolescents with cancer.

### mHealth Care Solutions for Adolescents

In response to this need, our team aimed to develop a smartphone-based pain app capable of providing adolescents with real-time pain management support. It is intended that this app will enable adolescents to better manage their cancer pain in real-time and in their natural settings. This app will use the PainSquad app our group previously developed [19] as an assessment platform. Specifically, we intend to build upon the PainSquad app by providing adolescents with cancer real-time pain management advice based on their PainSquad pain assessments.

At present, smartphone apps have been designed for and tested in a variety of health conditions and with a variety of patient populations. Conditions that have been targeted by apps include diabetes, alcoholism, cancer, and post-traumatic stress disorder [19-24]. A consistent focus of researchers to date has been on the development of apps to aid in patient self-management of chronic diseases or symptoms. In this capacity, apps may support patient self-monitoring, provide self-care advice, or facilitate interaction with health care providers and peers. Because mHealth is a relatively burgeoning field, few rigorous examinations of the impact of smartphone app interventions on patient, provider, and system outcomes have been conducted, especially in the area of pain [25]. Still, the application of patient-centered e-interventions to health problems has been evaluated in both adults and children in the form of computer-based Internet programs. Developed programs have been created for patients with conditions including arthritis, depression, anxiety, alcoholism, diabetes, and migraine [26-33]. These programs have positive effects across a range of health outcomes related to health status, health-related quality of life, symptom management, and disease knowledge. Further highlighting the potential for positive outcomes related to e-based programs, high rates of adherence with the interventions, low rates of study dropout, and high ratings of intervention acceptability have been observed [27,28,32,34,35].

To maximize the clinical utility and effectiveness of the new pain management app, our group intended to develop evidence-based and clinically appropriate care tools [36]. Specifically, as a pain care tool, an algorithm to guide pain care advice given by the app in response to particular pain inputs was considered to be especially advantageous. Algorithms are readily adaptable for use as part of computerized systems because they include explicit decision points [37] and help to ensure quality care by standardizing clinical decision making [38-40], while also allowing for individualization if guided by a patient’s data. The effectiveness of algorithm-informed and e-based cancer symptom monitoring and management has also been demonstrated. Research in the area in adults with cancer has demonstrated >30% decreases in symptom occurrence when care is compared to usual medical regimens [41]. A set of system requirements (eg, key functionalities and features) needed to improve the clinical effectiveness of the app was also considered to be important. These requirements may aid in improving the acceptability of the app to end users, or improve the ability of the app to fit with an institution’s policies and workflow [36].

### Approaches to the Development of mHealth Interventions

There are inherent challenges (ie, time and resource utilization, need for safe and effective interventions) to the design and eventual evaluation of complex health care interventions. To address these challenges, a phased approach to intervention design and testing has been encouraged [42]. The United Kingdom Medical Research Council (UK MRC), specifically, has proposed a framework to guide the development of well-utilized and effective complex interventions for health care [42,43]. The UK MRC approach can be used by mHealth developers throughout the app design and evaluation phases to minimize resource utilization and maximize potential effectiveness.

In addition, user-centered methods to the design of complex interventions, such as apps, have been repeatedly advocated for in the literature. User-centered design is an approach that grounds the process of product design in the needs and understandings of end users [44-46]. Incorporating stakeholders in all stages of the design of complex interventions means that the resultant products are more likely to be used by end users as intended, which will affect intervention effectiveness [44,45,47]. Critiques of user-centered design, including issues navigating the complex dynamics of multidisciplinary teams, time and financial costs associated with development, have been made [45,47]. However, to date, several studies that have incorporated user-centered design in the development of mHealth interventions have shown the resultant interventions to be well used and/or effective when tested [19,20,36].

Direct quantitative comparisons of products designed using a user-centered approach and those designed using more traditional developer-centered approaches on product usability are now beginning to be conducted. Results of these comparisons will be important in providing further evidence for the usefulness of user-centered design methods. For instance, the efficiency, usability, and safety of a computerized physician order set developed using user-centered design methods has been compared to that developed by the hospital informational technology team [48]. Results indicated that the order set designed with physician input from the onset was significantly less time consuming to complete compared to that designed by computer specialists. When using the order set designed with user-centered principles, physicians also requested significantly less assistance from the research team than when trialing the system designed by computer specialists.

In this paper, the development of clinical tools that will inform pain management advice given by a smartphone app is described. The specific objective of this research was to use a phased-approach (2-day consensus conference and iterative interviewing with system end users) to build, vet, and refine a pain care algorithm and catalogue of system requirements for the adolescent cancer pain management app.

## Methods

### Phase 1: Multidisciplinary Consensus Conference

#### Overview and Planning

A 2-day multidisciplinary conference in Toronto, Canada was held to develop consensus regarding the important pain inputs, pain management advice, and feature requirements of the mobile phone-based pain management system for adolescents with cancer. The content and structure of the conference was decided upon using modifications to the methods of a previous and successful health care consensus conference [49]. A facilitator for the meeting with research and clinical expertise in pediatric pain and pediatric oncology, as well as expertise in consensus conference methods and facilitation, was selected to lead the proceedings. Research Ethics Board approval was obtained at the host institution.

A systematic review of the published pediatric oncology pain management literature was conducted prior to the meeting to understand the current body of knowledge pertaining to pediatric cancer pain management. Results of this review, along with the meeting agenda, were disseminated to conference participants in advance of the meeting for their review and consideration.

#### Participants

Fifteen participants were selected based on their professional and research expertise in pediatric pain, pediatric oncology, or mHealth technology development and testing. Health care experts provided input on the needed clinical aspects of the system, and technology experts provided input on the technical practicality and feasibility of the system suggestions made by clinicians. A consumer (ie, a university educated adult living with chronic pain) was also included. The pain consumer did not have cancer but was asked to participate because of their extensive experience with pain and participation in scientific research and clinical projects. Table 1 outlines the demographic characteristics of conference participants.

#### Process

This conference used nominal group technique to develop consensus on the necessary features of the smartphone-based pain management system. Nominal group technique is a method used to pool judgment from a group of subject matter experts through quantitative and qualitative data collection methods [50-52]. Briefly, nominal group technique involves the use of a highly structured face-to-face meeting consisting of 2 facilitated rounds of voting on solutions to a problem posed to the group [51,53]. In the consensus conference conducted for the present study, an introductory presentation to conference participants on Day 1 highlighted (1) results of the systematic literature review, (2) overall goals of the 2-day meeting, and (3) reviewed the nominal group method. When the proceedings began, a question was posed to the group (eg, “What are the ‘patient-driven’ pain treatment techniques adolescents may use to manage their pain?”). A list of all questions posed to the group is shown in Multimedia Appendix 1. Each participant then had 2 uninterrupted minutes to address the group with 1 particular answer to the question and their rationale for selecting this answer. Answers were recorded and participants voted on the validity of each answer. Results of the vote were presented to the conference participants by the facilitator and each participant had an additional opportunity to address the group regarding the selected answers. A second round of voting followed. Consensus was considered achieved when 75% of the participants endorsed a given answer. All answers that did not reach 75% endorsement were discarded or reformulated through discussion. Conference proceedings were audio-recorded and field notes were made during both days.

Following the consensus conference, participant answers to the posed questions were used to develop a catalog of system requirements and a standardized pain treatment algorithm. When clarification on the data generated during the conference was required, consensus conference participants were queried by email and provided feedback. Audio-recordings and field notes were also referred to as necessary.

**Table 1:** Consensus conference participant characteristics (N=15).

Characteristic		n (%)
**Expertise**		
	Pediatric oncology clinician	7 (57)
	Pediatric pain clinician	4 (27)
	mHealth software design	3 (20)
	Pain consumer	1 (7)
**Country of residence**		
	Canada	9 (60)
	United States	4 (27)
	Australia	1 (7)
	Sweden	1 (7)
**Highest degree/licensure obtained**		
	MD PhD	3 (20)
	RN-EC PhD	3 (20)
	MD	2 (13)
	PEng PhD	2 (13)
	PhD Psych	2 (13)
	BA	1 (7)
	BASc MBA	1 (7)
	MN RN-EC	1 (7)

### Phase 2: Algorithm Vetting and Refinement

#### Overview

Prior to commencement of Phase 2 of this study, Research Ethics Board approval was obtained from the participating institution. A catalogue of system requirements and a prototype algorithm were developed based on Phase 1 results and iterative cycles of individual qualitative interviews were conducted with pediatric oncology and pain health care providers and adolescents with cancer to vet and refine the algorithm and requirements. A computer-based functional mock-up of the app was also developed to test with participants to improve their understanding of the intervention under development and focus their recommendations.

#### Participants

Participants were recruited from 1 large pediatric academic health care institution (Toronto, Canada) over a 3-month period in 2013. Health care professionals were included in the study if they specialized in either pediatric pain or oncology, were English speaking, were from any clinical profession, and had worked for at least 1 year. Health care professionals were excluded if they were in training and had not achieved full licensure. Adolescents who were English speaking, 12-18 years of age, diagnosed with cancer at least 3 months before testing, actively undergoing cancer treatment, and having experienced pain of any intensity in the week preceding testing were eligible to participate. The adolescent exclusion criterion was severe cognitive impairment as identified by a member of their health care team. A convenience sampling strategy, augmented with purposive sampling, was used to attempt to achieve maximum sample variation in age, sex, and diagnosis for adolescents. Maximum variation in profession was sought from health care professionals. This sampling strategy provided information-rich cases and ensured insight from a heterogeneous sample. Characteristics of both groups are shown in Table 2.

**Table 2:** Qualitative interviewing participant characteristics.

Characteristic			n (%)
**Health care professional participants (N=9)**			
	**Expertise**		
		Oncology	7 (78)
		Pain	2 (22)
	**Profession**		
		Physician	5 (56)
		Nurse	1 (11)
		Nurse practitioner	1 (11)
		Physical therapist	1 (11)
		Psychologist	1 (11)
	**Highest degree/licensure obtained**		
		MD	3 (33)
		MD MSc	2 (22)
		MScN RN-EC	1 (11)
		PhD Psych	1 (11)
		PT MSc	1 (11)
		RN BScN	1 (11)
**Adolescent participants (N=10)**			
	Age in years, mean (SD, range)		14.9 (2.0, 12.0-17.8)
	Years since diagnosis, mean (SD, range)		1.2 (1.6, 0.3-5.0)
	**Sex**		
		Female	3 (30)
		Male	7 (70)
	**Diagnosis**		
		Lymphoma	4 (40)
		Acute myeloid leukemia	3 (30)
		Ewing’s sarcoma	1 (10)
		Osteosarcoma	1 (10)
		Wilm’s tumor	1 (10)
	**Smartphone ownership**		
		No	1 (10)
		Yes	9 (90)
	**Comfort with smartphones**		
		Not at all comfortable	0 (0)
		A little comfortable	0 (0)
		Comfortable	1 (10)
		Very comfortable	9 (90)
	**Smartphone use per day** ^a^		
		Less than 7 times	1 (11)
		7-10 times	3 (33)
		Greater than 10 times	5 (56)

^a^Data are for adolescents who own smartphones only (N=9).

#### Process

Audio-recorded individual interviews were conducted with adolescents with cancer and health care professionals. Interviews were conducted with each participant group until data redundancy or the point when no new data were gathered that had not previously been categorized [54]. Each adolescent participant completed brief demographic and disease-related questionnaires, as well as a survey related to smartphone use. Additional information was obtained from medical charts as needed. Each interview lasted between 20 and 45 minutes. Semistructured interview guides were used to lead the discussion. Participants were given a brief description of the app and its intended function. Adolescent participants were additionally shown a semifunctional mock-up of the app using laptop-based smartphone emulator software. Very early in the interview process, it became clear that adolescents were not comfortable commenting on and critiquing the developed algorithm. As such, the process of algorithm vetting was conducted only with health care professionals because they are accustomed to algorithm use in the clinical setting and could provide valuable clinical insight.

Interview questions moved from general to specific and focused on eliciting adolescent and health care professional perceptions and suggestions on the system and algorithm [55,56]. The interview guides used with both adolescent and health care professional participants are shown in Multimedia Appendix 2. The interviewer recorded field notes on participant comfort with the interview process and other nonverbal communication. All audio-recorded interviews were transcribed and entered into NVivo 10.0 software [57] for coding. Field notes were also transcribed and coded alongside transcripts using NVivo 10.0.

#### Analysis

The qualitative data analysis process began as soon as the first interview was conducted. Two people independently conducted data analyses and discussed themes that emerged from the interviews with reference to field note data. Discrepancies in opinion were resolved using group discussion with a third party. A conventional qualitative content analysis approach was used to code data [58]. Specifically, each analyst independently made notes on impressions and thoughts (codes) related to the interview, according to the study objectives [55]. Codes were then grouped into meaningful categories or themes that reflected perceptions and suggestions for the smartphone-based pain management system and its clinical algorithm. Changes to the catalogue of system requirements and the algorithm were made following individual interviews. Further interviews were conducted until neither the adolescents nor health care professionals had further suggestions for changes. All descriptive quantitative data (for both study phases) were analyzed using SAS 9.1 software [59].

## Results

### Phase 1: Multidisciplinary Consensus Conference

#### Overview

The 2-day consensus conference established the key elements of the smartphone pain management system, which could be used to build the catalogue of system requirements and design the preliminary pain care algorithm.

#### Clinically Important Pain Inputs by Adolescents

Clinically important pain inputs defined the clinical characteristics of an adolescent’s pain experience that should result in pain management support from the app. Specifically, a clinically important pain input represented a threshold of adolescent-reported pain that facilitates the generation of automated help. Using the nominal group technique, 11 pain inputs were originally suggested by clinical experts. Following voting and a second round of discussion, 4 inputs were retained as thresholds for system-delivered pain management advice. These adolescent inputs were (1) pain of any severity now, (2) pain of any severity in the previous 12 hours, (3) pain interference with activities of daily living, and (4) a sense of not being able to control pain. Below, a clinical psychologist discussed the rationale for including multidimensional pain assessment responses as important inputs:

“We’ve talked a lot about assessing the intensity of pain…but from the psychology side one of the missing ingredients is the interpretation of pain in some way…So, it becomes important to understand how someone is coping with pain and the emotional climate when pain is occurring…because pain interpretation is an important part of self-management techniques.”

#### Appropriate Pain Management Advice From the System

The appropriate pain management advice, which the smartphone system could provide to adolescents, was also established. Pain management advice was grouped as being pharmacological, physical, or psychological in nature. Endorsement for this grouping is illustrated in a quote from a pediatric pain management expert:

“I agree that you need the broad categories up front. And then, you know, you may…click on a selection and you have everything within it and you can delve in a bit deeper for some suggestions about things you might use.”

Clinical experts concentrated their suggestions on pain management advice that was considered appropriate for an adolescent to undertake on their own. Therefore, complex pain management strategies, or strategies requiring advanced clinical training and licensure (eg, medication adjustments), were not suggested as potential advice for adolescents.

Consensus conference participants suggested 2 pharmacological options, which were retained throughout voting. Pharmacological options were reminders to take prescribed medications. Six physical strategies were suggested and 4 were retained after both rounds of voting, including suggestions such as the use of hot/cold therapies. Eighteen psychological pain management therapies were originally suggested, with 9 being retained after voting. Psychological recommendations included using distraction and seeking social support, among others.

#### System Requirements of the Smartphone-Based Pain Management System

Finally, nominal group technique established the needed system functionalities to maximize the effectiveness of the app. Following 2 rounds of voting, 5 requirements were retained and included in the smartphone system design. Table 3 details the system requirements and the rationale for including each in the pain management system.

**Table 3:** Requirements of the smartphone-based pain management app based on expert consensus

Design feature	Feature details	Rationale for feature	Representative health care professional quote
Truncated ad hoc pain assessment	In addition to scheduled long-form morning and evening pain assessments, adolescents will have the ability to complete a truncated (short-form) pain assessment on an ad hoc basis.	The ad hoc assessment will provide the ability to complete a pain assessment and receive timely, ‘in-the-moment’ pain management advice during pain episodes. A truncated assessment for ad hoc reports will minimize response burden for adolescents.	*If [kids] miss that morning window to complete a report, what happens? They should be able to log pain when they have pain.*
Delivery of multiple different pain management recommendations	Adolescent-logged pain data will drive the app’s provision of a list of several user-selectable pain management recommendations. The pain management algorithm will determine the generated list.	The provision of several different pain management recommendations will minimize the chance that an adolescent is provided with undesirable or inconvenient advice. Note: The system will recognize “pain emergencies” (eg, sustained severe pain based on historically saved survey records) and recommend emergency action.	*And I think we should take into consideration your point…and make sure that we give [a variety of therapies] that work over a variety of training and developmental stages.*
Access to a selected pain management recommendation	Upon selection of a pain management recommendation, adolescents will be able to access this pain management strategy via the app (eg, if ‘listen to music’ is selected, the app will link available music on the phone).	Direct access to the pain management strategy will minimize time-to-intervention and will maximize automation of tasks to improve the user-experience. Note: Direct access will not be available for all recommendations (eg, talk to your parent).	*We could also think about, you know, ‘are there videos or something that you could embed in the app?’ Because that app could be really good at providing [access to] those types of things.*
Pain re-assessment	Pain will be re-assessed 1 hour following a pain management recommendation.	A follow-up assessment should be conducted to assess pain after the management recommendation. If an adolescent remains in pain, another recommendation should be made.	*Is there some capacity for a reminder alarm for a re-assessment. So that you learn in real-time how effective each of these things are.*
Capacity to prevent or mitigate procedural pain	Adolescents will have the ability to inform the system of upcoming painful procedures (eg, venipuncture) and receive advice on pain prevention or mitigation strategies (eg, ‘remember to listen to a favorite song’).	To be as comprehensive as possible in managing adolescent cancer-related pain, the app should endeavor to prevent and mitigate procedural pain.	*A kid’s going in for a procedure sometime in the future…we’re going to have a toolbox that’s ready for procedures. And the question is what’s in that toolbox for something like a finger-stick all the way up to something more invasive.*

### Phase 2: System Refinement and Algorithm Vetting

#### Overall Endorsement of the System

All participants endorsed the smartphone system and thought that it would be beneficial to adolescents with cancer pain. Specific beneficial aspects of the system from the point of health care professionals included the ability to manage pain the moment it occurs and to intervene when adolescents experience cancer pain in their home environments. The benefits of the system as cited by adolescents included having a record of pain and pain care to discuss with their health care team at subsequent clinic visits, the ability to connect through the system with other adolescents with cancer, and the ability to manage pain the moment it occurs, including through the use of several psychological and physical management strategies. Adolescent endorsement of the ability to receive several diverse pain management strategies is highlighted in the quote below.

“[I like] that there’s different options available. It’s not like, ‘Ok, take this medication or something’. Yeah, I think I like that about the app…Like, you can learn more about pain and…you know, you could like write a journal too.”

#### Addition of a Clinical Expert

Overwhelmingly, both oncology health care professionals and adolescents endorsed the inclusion of a clinical expert in the smartphone system design. The app was originally designed as a stand-alone system that would provide pain management advice to adolescents without the active input of a health care professional. However, following the interview process it was decided that the active input of a clinical expert (eg, an oncology-trained registered nurse) would improve both the effectiveness and safety of the system. The system design was then changed so that adolescent pain reports considered clinically important (eg, 3 consecutive reports of moderate-to-severe pain [pain ≥ 4/10]) will trigger an email alert to a registered nurse. The nurse will then contact the adolescent to assist in clinical decision making. A representative quote from an adolescent about the addition of a health care professional is below:

“Um, if [a health care professional] contacts you, I think that also makes sense*.* But, um, like I’m not sure if you need them to contact for every single pain. But, I think if it falls under certain categories then that’ll be good.”

#### Individualization of the System

Both health care professionals and adolescents recognized that individualization of pain management advice is important. Both groups highlighted that individuals may respond to pain management advice uniquely based on their personal characteristics and preferences. One adolescent stated:

“I don’t know, I think it depends on the person, I think. Like, I wouldn’t mind it talking to my parents, but that’s just me I guess.”

As such, a mechanism that allows adolescents to rate the likability and effectiveness of the pain management advice the system offers was added to the design. Through this mechanism, when future pain management advice is given to adolescents, the advice adolescents liked and used most often will be offered first. A capacity for the system to individualize pain management advice to a specific adolescent was endorsed by health care professionals as highlighted in the below quote:

“You can kind of think about this as an N of 1 trial each time somebody is doing it. So if there is a way to build in that learning, then ultimately [the app] becomes evidence-based for that particular person.”

#### Vetting of the Algorithm With Health Care Professionals

Vetting of the algorithm occurred with health care professionals who recommended changes to the algorithm based on their clinical and research expertise. Required major changes included revision of the threshold for a change in pain management, suggestion that the wording used in app be changed to improve an adolescent’s ability to understand questions and management advice, and the recommendation that adolescents be informed regarding time to contact from the registered nurse monitoring the system. For instance, a health care professional said this regarding the wording used in this app:

“This could be clearer in terms of the language here. It’s almost like in needs to be black and white, ‘take your narcotic’.”

In response to these required changes: (1) the threshold for the system to offer additional advice was lowered so that if pain was not improved 1 hour after initial management advice was made, new advice may be delivered; (2) adolescent-appropriate wording of system questions and management instructions was used; and (3) an alert was added to the system to tell adolescents that a health care provider was being informed about their pain. The alert further stated that if the adolescent did not hear from the health care professional within a given amount of time, they should talk to their caregivers and/or seek medical help. Following these revisions, clinicians suggested no further changes and the algorithm was considered finalized.

## Discussion

### Principal Findings

The pain decision algorithm and catalogue of pain management system requirements were successfully developed using a 2-day consensus conference with clinical and technology experts, and an iterative interview process with system end users (ie, health care professionals and adolescents with cancer). Through the methods presented here, we were able to establish core pain inputs from adolescents, appropriate pain management advice, and necessary system requirements for a remote real-time pain management system.

The stepwise approach endorsed by the UK MRC has provided an important framework for the development of the smartphone-based system and its algorithm. The systematic review conducted before the consensus conference enabled understanding of the current scientific evidence regarding pain management in adolescents with cancer. The review was required to provide the necessary data for the development of a system that delivers cancer-specific evidence-based pain management support to adolescents. The 2-day consensus conference provided data that were synthesized to develop the pain management algorithm that is fundamental to effective system performance. The use of nominal group technique at this conference successfully enabled the pooling of thought and opinion from a highly diverse group of experts. Qualitative interviewing with both adolescents and health care professionals then provided critical feedback regarding their perceptions of system and algorithm utility and safety, as well as suggestions for improving overall design and function. Interview data can now be used to inform the user-centered design principles for the development of the app software [46].

Internet- and mobile-based interventions to manage symptoms of diseases such as cancer have the potential capacity to provide patients with cost-effective, accessible, and high-quality treatments [29,60-62]. Because of these perceived benefits, using e-based health interventions as the initial step in a stepped-care approach to patient care has been suggested [29]. A stepped-care approach as a model for care describes the initial provision of a form of care to all patients that is typically cost-effective and low intensity. Care is then escalated for patients who do not sufficiently benefit clinically from the initial intervention. On the other hand, a stratified model to the delivery of care, including e-based care, may be used. In this case, the selection of an initial intervention is based on an a priori assessment of a given patient.

Lipton et al [31] conducted a randomized trial to assess the effect of each of the stepped-care and stratified models on headache pain intensity and disability in a group of adults. Results showed that patients stratified to care based on headache disability at study onset had a significantly better response to treatment than those treated using a stepped care approach. With respect to the adolescent pain management app in question, the effectiveness of the intervention is likely to vary depending on an adolescent’s pain and their social, psychological, and demographic characteristics. A stratified approach to delivering the app as a health care intervention may therefore be warranted. However, the criteria by which to allocate adolescents to a given strata remains to be established.

### Comparison With Prior Work

The methodology used in this study has been used previously to develop care algorithms and assessment tools. For instance, using qualitative interviewing with clinical experts and iterative refinement, a rule-set for the remote telemonitoring of patients with heart failure was developed [36]. The monitoring system based on the rule-set was tested in a randomized control trial, which showed the preliminary effectiveness of rule-set-based e-systems [63]. Nominal group technique has also recently been used to develop consensus on the needed elements of an electronic symptom assessment questionnaire for children with cancer [64]. The authors of this study were able to develop a preliminary version of a new assessment tool and attributed a part of the success to the use of nominal group technique. Finally, similar consensus conference methodology has been used to develop an e-based pain assessment questionnaire for use in routine pediatric rheumatology [49], which is currently undergoing validity and reliability testing.

The finding that the inclusion of a clinical expert in the system design was recommended as an important means to improve system effectiveness is consistent with previously published research. For instance, the importance of clinician involvement has been shown in a 2012 systematic review of the content, structure, and efficacy of pain self-management programs for adults with cancer [65]. In this review, those studies with the largest effect sizes in terms of pain reduction included the interaction between clinicians and study participants. Second, in a meta-analysis of Internet-based cognitive behavioral therapy for depression and anxiety, interventions that included therapist support were more effective than those without. Specifically, interventions with support had a large pooled mean effect size (1.0; 95%CI, 0.75-1.24), while those without support had a small pooled mean effect size (0.26; 95%CI, 0.08-0.44) [26]. Together, these results suggest that interventions across a variety of symptoms and diseases may benefit from the inclusion of a clinical expert. Furthermore, although the optimal of dose of clinician contact in Internet- and mobile-based interventions has not been established [61], these results also provide support for the inclusion of such an expert in the pain management app discussed presently.

### Limitations

The results presented in this study are tempered by limitations that should be addressed. For instance, although we used qualitative interviewing with adolescents to vet and refine the algorithm, we did not include patients or families in the consensus conference. The inclusion of adolescents with cancer and their parents would have provided valuable insight into end-user perceptions of system and algorithm utility that could have streamlined the interviewing process. An additional limitation is that the developed algorithm and list of system features were developed through qualitative interviewing at 1 hematology/oncology center. The smartphone-based app with health care provider support described here is therefore optimized to the clinical practices and workflow of this center. Adolescents with cancer who are from multiple centers will be enrolled in feasibility testing of the pain management app, which will afford the opportunity to examine the practicality of our system across diverse care environments.

### Conclusions

The use of a phased user-centered approach used in the present study allowed for the efficient and successful development of the decision algorithm and smartphone system design. The methodology presented here may represent a viable roadmap for the creation of e-based health systems and care algorithms for a variety of health conditions and populations. Next steps will involve development of the real-time pain management app interface as well as usability and feasibility testing of the system with adolescents. Successful development of this smartphone-based pain management app has the potential to improve pain management for adolescents with cancer, minimize barriers to optimal symptom treatment, and enhance interaction with health care providers to improve quality of life. Moreover, the model of technology-assisted care represented by this app has the potential to usher in a paradigm shift in the care of patients with a variety of chronic and life-threatening illnesses. If ultimately effective, this app as a care model represents an excellent opportunity to improve patient care through remotely monitoring and efficiently managing a range of physical and psychological health conditions. 

## Multimedia Appendix 1

Voting questions for consensus conference.

## Multimedia Appendix 2

Guide for individual algorithm vetting and refinement interviews.

## Abbreviations

UK MRC: United Kingdom medical research council
