# Supplementary material for: A Smartphone-Based Pain Management App for Adolescents With Cancer: Establishing System Requirements and a Pain Care Algorithm Based on Literature Review, Interviews, and Consensus
Source: JMIR Res Protoc. 2014 Mar 19;3(1):e15. doi: 10.2196/resprot.3041 (PMC3978558; doi:10.2196/resprot.3041)
Supplement: Supplementary file 1 [file resprot_v3i1e15_app1.pdf]

Appendix 1. Voting questions for consensus conference

Question

1. What inputs or combinations of inputs should trigger a pain management from the app to the adolescent (e.g. Persistent pain for x hours/days? Pain spreading through body? Sudden onset pain? High interference scores? Specific interference elements like low mood, poor sleep? Pain management techniques not working?)
2. What are the 'patient-driven' pain treatment techniques adolescents should use to manage their pain?
3. Which patient-driven pain management techniques (including dosage and frequency) should be used to manage:
    - Treatment-related pain with intensity 1-3/10
    - Treatment-related pain with intensity 4-6/10
    - Treatment-related pain with intensity 7-10/10
4. Which patient-driven pain management techniques should be used to manage:
    - Disease-related pain with intensity 1-3/10
    - Disease-related pain with intensity 4-6/10
    - Disease-related pain with intensity 7-10/10
5. How should procedural pain be reduced by the adolescent in the case of a:
    - Port-access?
    - Lumbar puncture?
    - Bone marrow aspirate?
    - Venipuncture?
    - Finger-poke?
