# Supplementary material for: A Smartphone-Based Pain Management App for Adolescents With Cancer: Establishing System Requirements and a Pain Care Algorithm Based on Literature Review, Interviews, and Consensus
Source: JMIR Res Protoc. 2014 Mar 19;3(1):e15. doi: 10.2196/resprot.3041 (PMC3978558; doi:10.2196/resprot.3041)
Supplement: Supplementary file 2 [file resprot_v3i1e15_app2.pdf]

Appendix 2. Guide for individual algorithm vetting and refinement interviews

Questions for adolescent participants

1. What do you like **best** about the idea of this app? What do you like **least** about the idea of this app?
2. Do you predict any difficulties with using the app (e.g. smartphone problems, not understanding the app questions, not knowing how to do the things the app recommends)?
3. What sort of pain management help should the app give you?

   Probes: Should the app help you make choices about when to take medications? Should the app help you make choices about doing physical things like exercising? Should the app help you make choices about doing things like relaxing, mediating and distracting yourself?
4. Now that you have an idea of the app we want to design, do you have any suggestions for the app?

   Probes: What types of things would the app have to have for you to want to use it?
5. Is there anything else you would like to mention about cancer pain management or this app?
6. What are your impressions of this plan detailing how the app will manage your pain (e.g. algorithm)? How should it be changed? How should it be improved?

Questions for healthcare professionals

1. What do you like **best** about the idea of the app? What do you like **least** about the idea of the app?
2. Do you predict any difficulties with using the app (e.g. smartphone problems, not understanding the app questions, not knowing how to do the things the app recommends)?
3. How large should the role of adolescents be in managing their own pain? When should healthcare professionals be involved? When should parents be involved?

   Probes: How often and how severe should pain be before the healthcare team needs to intervene and assist?
4. How should healthcare professionals be alerted to potentially serious pain reports logged by adolescents or situations when adolescents may need assistance in managing their pain?

   Probes: should the app automatically inform the team? Should the adolescent or parent be responsible for contacting the team?
5. What is the perceived clinical utility of the CDSS from the perspective of the healthcare professionals and how could utility be enhanced?
6. If a fully functional iPhone app was created and in use by adolescents with cancer, how do you see responding to alerts and reviewing data fitting into your daily workflow?

   Probes: How might this app help and/or add to workflow? How could this be minimized or optimized?

7. What are your impressions of this plan detailing how the app will manage your pain (e.g. algorithm)? How should it be changed? How should it be improved?
